# Theory of Mind and Emotional Awareness in Chronic Somatoform Pain Patients

**DOI:** 10.1371/journal.pone.0140016

**Published:** 2015-10-07

**Authors:** Matthias Zunhammer, Agnes Halski, Peter Eichhammer, Volker Busch

**Affiliations:** 1 Neurologie, Lehrstuhl für Neuroimaging, Universitätsklinikum Essen, Hufelandstraße 55, 45147 Essen, Germany; 2 Klinik für Psychiatrie und Psychotherapie, Universitätsklinikum Regensburg, Universitätsstr. 84, 93053 Regensburg, Germany; Iranian Institute for Health Sciences Research, ACECR, ISLAMIC REPUBLIC OF IRAN

## Abstract

**Objective:**

The present study aimed at investigating whether chronic pain patients are impaired in Theory of Mind (ToM), or Emotional Awareness.

**Methods:**

Thirty inpatients suffering from chronic somatoform pain, as well as thirty healthy controls matched for age, sex, and education were recruited. ToM abilities were measured using the Frith-Happé animation task, in which participants interpret video-clips depicting moving geometric forms that mimic social interactions. The responses given were scored for appropriateness and the degree of inferred intentionality according to established protocols. Emotional awareness was measured using the Levels of Emotional Awareness Scale (LEAS), for which participants provide written descriptions of feelings in imaginary emotional situations. Standardized scoring was performed to capture the number and quality of emotional terms used.

**Results:**

Responses lengths were similar in both groups and for both tasks. Patients attained significantly lower intentionality but not appropriateness scores when interpreting ToM interactions. No significant group differences were found when interpreting goal directed interactions. Emotional awareness scores were significantly lower in patients compared to healthy controls.

**Conclusions:**

Our results suggest that chronic pain patients are impaired in mentalizing and emotional awareness. Future studies are needed to determine whether these ToM and emotional awareness deficits contribute to the etiology of somatoform pain and whether addressing these deficits in therapeutic interventions can improve polymodal pain therapy.

## Introduction

Theory of Mind (ToM), or mentalizing, is the ability to infer on mental states, such as intentions, beliefs, or emotions in others [1]. Two studies recently suggested that patients with somatoform disorders may be impaired in ToM [2,3]. Both studies used the Frith-Happé-Animation Task (FHAT) as a measure of ToM [1] and authors of both studies suggested that ToM-deficits might impair proper mental representation of emotions, in turn leading to somatoform symptoms.

The ability of patients with somatoform disorder to label and represent emotions has been investigated for several decades: A wealth of literature indicates that somatoform disorders are associated with alexithymia, the inability to identify and label emotions [4]. However, the validity of the main measure of alexithymia, the Toronto Alexithymia Scale (TAS), has been questioned [5] and it has been proven difficult to establish a causal relationship between alexithymia and somatization in longitudinal studies [6,7]. By using the “Levels of Emotional Awareness Scale” (LEAS) [8], a performance based measure of emotional awareness, Subic-Wrana et al. could show that somatoform patients use fewer emotional terms than other psychiatric inpatients [9] and healthy controls [2]. Two other studies using the LEAS did not find such emotional awareness deficits when comparing somatoform patients with healthy controls [10] and patients with medically explained symptoms [3].

The aim of the present study was to follow up these interesting findings and to extend the existing knowledge by focusing on chronic somatoform pain, the most prevalent sub-form of somatization disorder [11]. Chronic somatoform pain is characterized by distressing but medically unexplained pain in combination with abnormal beliefs and behaviors regarding the pain, with a minimum duration of 6 months. We aimed at investigating ToM and emotional introspection in chronic pain patients in comparison to a healthy control group matched for sex, age, and education. We hypothesized that chronic pain patients would show lower ToM abilities, as measured by the FHAT and lower emotional awareness, as measured by the LEAS.

## Methods

The present study was approved by the ethics committee of the University of Regensburg (Approval Number: 08/132) and conforms to the Declaration of Helsinki [12]. Written informed consent was obtained from all participants.

### Subjects

Thirty inpatients of the Clinic for Psychiatry and Psychotherapy at the University of Regensburg were recruited. Main inclusion criterion was the diagnosis “Pain Disorder associated with Psychological Factors” according to DSM-IV Code 307.80. An experienced physician performed the diagnosis and the evaluation of exclusion criteria, using the Structured Clinical Interview for DSM-IV (Axis I) [13].

Thirty healthy controls were recruited successively and matched for sex, age, and education. Exclusion criteria for both groups were: major internal or neurological disorders, organic and inflammatory pain disorders, schizophrenia, major depression, current use of sedative medication, and insufficient German language skills. Exclusion criteria were evaluated in a structured interview. For the control groups the short-version of the Structured Clinical Interview for DSM-IV (Axis I) was used for screening, in order to exclude any mental disorders [13].

### Material

#### The Frith-Happé-Animation-Takss

The Frith-Happé-Animation task (FHAT) is a performance measure of ToM, based on animated video-clips of 34 to 45 seconds, in which two triangles exhibit the movement patterns of living beings [1]. Healthy individuals automatically identify objects showing such movements as intentional agents [14]. The FHAT therefore allows probing participant’s ability to recognize ToM in interpersonal interactions, while excluding most of the potentially confounding cues and contexts typically present in social settings. The FHAT has been successfully applied to detect impaired ToM abilities in autistic children [1] and depressive patients [15].

The FHAT comprises four video-clips, in which two triangles display interactions suggesting „Surprising“, „Coaxing“, „Mocking“, and „Seducing”behavior—all of which require ToM for correct interpretation (ToM-condition) [1]. As a control, the FHAT includes four further Goal-Directed video-clips (GD-condition), in which two triangles display interactions suggesting „Fighting“, „Following“, „Chasing“, and „Dancing“, i.e. behaviors which do not require higher-order mentalizing abilities for recognition [1]. After each video-clip, participants are asked to describe what they observed. The responses are recorded and subsequently scored on the FHAT-intentionality and the FHAT-appropriateness scale [16]. The FHAT-intentionality score reflects to which extent deliberate actions and intentions are ascribed to the triangles on a 5-point scale (for details see: [16]): A score of 0 is given for a description of non-deliberate action (“bouncing”, “moving around”), whereas a score of 5 is given for a description of a deliberate action aimed at affecting another’s mental state (“The blue triangle wanted to surprise the red one.”) [16]. The FHAT-appropriateness scale is a measure of how close the given response matches the content of the video, as intended by the designers [16]. A score of 0 is given for no answer, or “I don’t know”, whereas a score of 3 is given for a correct interpretation (see: [1]).

#### The Levels of Emotional Awareness Scale

The Levels of Emotional Awareness Scale (LEAS) is a performance test [8], which comprises twenty scenes (vignettes), each describing an imaginary situation in which the participant interacts with a second person. Participants are asked to give a written description of the emotions the situation may invoke in them and in the second person involved [8]. Each response is rated on a five-point scale, corresponding to one of five hypothesized “levels of emotional awareness” [8,17]. The construct of emotional awareness is hereby defined as “*type of cognitive processing which undergoes five levels of structural transformation along a cognitive-developmental sequence derived from an integration of theories of Piaget and Werner*. *The five levels of structural transformation are awareness of 1) bodily sensations*, *2) the body in action*, *3) individual feelings*, *4) blends of feelings*, *and 5) blends of blends of feelings*.” [18]. Put simple, the LEAS has been proposed to measure the “ability to identify and describe emotions” [19].

#### Additional clinical measures

Symptoms in the patient group were characterized using the German Pain Questionnaire (“Deutscher Schmerzfragebogen”, GPQ) [20]. The GPQ, includes the SF-12, a questionnaire measure of quality of life [21]. Both patients and controls completed Beck’s Depression Inventory-II (BDI-II) [22] to quantify depressive symptoms and the German 26-item version of the Toronto Alexithymia Scale (TAS-26) [23] to quantify alexithymia.

### Procedure

After study inclusion, each participant was administered one of the two LEAS half-forms (LEAS A, LEAS B) at random. Each half-form comprises ten of the twenty LEAS items [17]. Participants were free to choose the length and style of response and were instructed according to the German LEAS manual (Subic-Wrana, unpublished). Subsequently, each participant was shown the eight FHAT-animations as used by Abell et al. (2000). The GD and the ToM video-clips were presented in mixed order. After each video-clip participants were asked: “What just happened in the cartoon?” No feedback, or help was given by the experimenter, except for general encouragement [1]. Participants’ responses were recorded using a video camera. The procedure was practiced once before the actual test, using a video-clip with randomly moving triangles [1]. Hereafter, participants were administered the remaining questionnaires.

### Analysis

Responses from both the LEAS and the FHAT were transcribed to written electronic form. The LEAS was scored according to the instructions and glossary for the German manual (Subic-Wrana, unpublished). FHAT-transcripts were scored on the “intentionality” and the “appropriateness” scale, according to [16].

Two investigators scored both the LEAS and the FHAT, independently: one (A.H.) had performed data acquisition and was not blinded in respect to the study group, whereas the other (M.Z.) was blinded to grouping conditions. Analyses and conclusions were based on the blinded rater’s score.

The within-subject scores for all GD and all ToM video-clips were pooled before further analysis. Mann-Whitney-U tests were used to determine potential group differences in all variables. Pearson’s correlation coefficient was used to as a measure of inter-rater reliability for both the LEAS and the FHAT. Word-counts for FHAT and LEAS responses were compared between groups and correlated with FHAT and LEAS scores to screen for potential motivational confounds.

Associations between LEAS, FHAT, TAS-26, BDI-II, current pain severity, and quality of life (SF-12), were tested using the non-parametric correlation coefficient Kendall’s tau-b. Means are reported ± SD if not stated otherwise. SPSS 21.0.0.0 for Mac was used for analysis.

## Results

All data are available in comma-separated format as S1 File. Mean age was 50.2 ± 8.6 in the patient and 47.2 ± 8.9 years in the control group. In both groups, the proportion of females was 67%. In both groups, the proportion of low, middle, and high educational status was 50, 30, and 20%, respectively.

Table 1 provides description of patient characteristics as obtained with the GPQ.

**Table 1:** Patient characteristics.

**Pain duration** (in years)	**n**
> 5:	15
2–5:	6
1–2:	6
0.5–1:	2
**No. of different pain domains** (e.g. abdominal pain, back pain)	**n**
1:	4
2:	9
> 2:	16
**Type of pain**	**n**
Constant with little fluctuations	3
Constant with strong fluctuations	17
Attacks with pain-free periods	2
Attacks with constant pain in-between	5
**Pain intensity**	**mean ± SD**
Mean rating on 101-pt visual analog scale	65.9 ± 14.3
**Physicians consulted for pain in the past**	**mean ± SD**
Mean number	5.2 ± 2.2
**Pain treatments/therapies in the past**	**mean ± SD**
Mean number	7.8 ± 4.2
**Current psychotropic/analgesic medication**	**n**
Selective serotonin re-uptake inhibitors / Serotonin–norepinephrine reuptake inhibitors	20
Non-steroidal anti-inflammatory	14
Opioid	10
Pregabalin/Gabapentin	6
Atypical antipsychotics	6
Tricyclic antidepressants	4
Agomelatine	2
Benzodiazepines	2
Lithium	1
Methylphenidate	1

Clinical parameters according to the German Pain Questionnaire. The number of subjects (N = 30) varies due to unanswered items.

### FHAT

The FHAT scores obtained by the blinded and the un-blinded investigator were strongly correlated for intentionality (r = .82) and for appropriateness scores (r = .86), denoting an acceptable inter-rater reliability. Intentionality scores were found to be significantly lower in patients, compared to controls in the ToM-, but not the GD-condition (Table 2). No significant group differences for the appropriateness scores were found—neither for the ToM-, nor the GD-condition (Table 2). Both FHAT intentionality (r = .402, p = .002) and FHAT appropriateness (r = .343, p = .006) scores were significantly correlated with FHAT word count, yet, FHAT word count did not significantly differ between groups (Table 2).

**Table 2:** Results for performance measures and questionnaires.

Scale	Patients (n = 30)	Controls (n = 30)	Mann-Whitney-U	p	Effect Size (Cohen’s d)
**Levels of Emotional Awareness Scale (LEAS)**					
Total	28.9 [26.4, 31.5]	32.6 [30.2, 35.1]	302.5	**.029**	**0.53**
Self	28.9 [26.4, 31.5]	32.0 [29.6, 34.4]	316.5	**.048**	**0.45**
Other	23.6 [20.7, 26.6]	28.5 [26.3, 30.7]	283.0	**.013**	**0.69**
Word count	172 [134, 209]	176 [139, 213]	433.5	.807	–
**Frith-Happé Animation Task (FHAT)**					
ToM-intentionality	14.9 [13.9, 15.9]	16.4 [15.5, 17.4]	317.0	**.048**	**0.56**
ToM-appropriateness	7.9 [7.4, 8.4]	8.1 [7.5, 8.8]	390.5	.369	**–**
GD-intentionality	12.2 [11.4, 12.9]	12.5 [12, 13]	390.5	.364	**–**
GD-appropriateness	10.2 [9.7, 10.7]	10.2 [9.6, 10.8]	429.5	.756	**–**
Word count	323 [230, 416]	290 [238, 341]	395.5	.703	
**Toronto-Alexithymia Scale 26-item (TAS-26)**					
Total	52.2 [48.4, 56]	41.1 [38.4, 43.8]	719.5	<.001	**1.21**
Difficulty identifying feelings	21.1 [19.1, 23.1]	12.3 [10.7, 13.9]	795.5	<.001	**1.76**
Difficulty describing feelings	15.6 [14.2, 17]	12.1 [11, 13.2]	676.5	.001	**1.03**
Externally orientated thinking	15.5 [14.2, 16.8]	16.7 [15.8, 17.6]	382.5	.32	–
**Beck’s Depression Inventory-II**					
Total	23.5 [20.2, 26.8]	5.3 [4.2, 6.5]	882.5	<.001	**2.94**

Means ± 95% Confidence Interval.

ToM: Set of video-clips displaying behaviors involving “Theory of Mind”. GD: Set of video-clips displaying goal-directed behaviors, not requiring “Theory of Mind”. Means ± SD.

### LEAS

The LEAS scores obtained by the blinded and the un-blinded investigator were highly correlated (r = .93), denoting a good inter-rater reliability. Patients had a significantly lower score on all LEAS sub-scales compared to controls; effect sizes were moderate (Table 2). LEAS total scores were significantly correlated with LEAS word count (r = .451, p = .001), but LEAS word count did not significantly differ between groups (Table 2). The scores obtained with the half-forms LEAS-A (30.6 ± 7.7) and LEAS-B (31.1 ± 6.2) did not differ significantly (U = 449.0, p = .63).

### Measures of depression and alexithymia

TAS-26 and BDI-II results indicate, that patients rated themselves as more alexithymic and depressed than controls (Table 2). Patients and controls differed on the TAS-26 sub-scales “difficulty identifying feelings” and “difficulty describing feelings”, whereas no difference in “externally oriented thinking style” was found. The differences in TAS-26 and BDI-II scores between groups had a large effect size.

### Associations between measures

Correlation analyses were used to explore the relationships between LEAS, FHAT, TAS-26, BDI-II, and clinical parameters within the patient group (see: Table 3). Quality of life, as measured by the to the SF-12, was associated positively with ToM intentionality-scores and negatively with TAS-alexithymia, BDI-depression, and VAS-ratings of clinical pain.

**Table 3:** Associations between measures of ToM, Emotional Awareness, and clinical parameters within the patient group.

Scale	1 LEAS total	2 FHAT ToM-intentionality	3 TAS-26 total	4 BDI-II	5 VAS-Clinical Painª
**1 LEAS total**					
**2 FHAT ToM-intentionality**	.081				
**3 TAS-26 total**	-.142	-.205			
**4 BDI-II**	-.167	-.180	.231		
**5 VAS-Clinical Pain** **ª**	.022	-.081	.254	.146	
**6 SF-12** **ª**	.192	.451**	-.389**	-.414**	-.194

n = 30. All values denote Kendall’s tau-b.

** p < .010.

^ª^ VAS-Clinical Pain and SF-12 scores obtained according to the German Pain Questionnaire [20]. VAS-Clinical Pain was calculated as the mean visual analog scale rating for the three items “current pain”, “mean pain during last 4 weeks” and “highest pain during last 4 weeks”.

## Discussion

### Further evidence for introspection deficits in chronic somatoform pain

The present results show that chronic somatoform pain patients make significantly fewer references to mental states when describing social interactions involving ToM. In contrast, patients did not differ significantly from controls when describing goal directed (i.e. non-ToM) interactions (FHAT-intentionality score, Table 2). For both types of social interaction, patients and controls did not differ significantly in their ability to correctly name the depicted interaction (FHAT-appropriateness score, Table 2). Our findings indicate that somatoform pain patients are subtly impaired in their mentalizing abilities. These results consolidate previous observations obtained in mixed somatoform samples [2,3].

Our study further replicates the finding that somatoform patients use significantly fewer words denoting emotional awareness when writing about emotional situations as compared to healthy controls (LEAS-results, Table 2) [2,9]. Together, these results indicate that chronic somatoform pain patients are impaired in inferring on mental states, as well as describing emotional states experienced by themselves and/or others. The fact that two previous studies did not find emotional awareness deficits in mixed somatoform samples using the LEAS [3,10] may be explained by differences in sample composition. For one, the two studies included different somatization sub-populations, such as, somatization disorder (ICD-10, F45.0) [10], somatoform autonomic dysfunction (ICD-10, F45.3) [10], somatoform pain (ICD-10, F45.4) [10], conversion disorder (DSM-IV, 300.11) [3,10], and functional somatic syndrome [3], while the present study focused on somatoform pain patients. Moreover, one of the studies [3] recruited outpatients in the US, while our sample consisted of in-patients in Germany. Therefore, systematic differences in socio-demographic features and disease severity may explain the observed differences in results.

### Potential causes and clinical perspectives

An exploration of associations between measures of introspection (LEAS, FHAT, TAS-26) and clinical parameters (BDI-II, VAS-pain, SF-12) yielded mixed results. Quality of life in patients was found to be correlated with FHAT intentionality score (see: Table 3), indicating that mentalizing abilities are associated with patients’ well-being. Although depressive symptoms were more prevalent in patients (see: Table 2), depression severity was not significantly correlated with FHAT and LEAS scores (see: Table 3). The observed introspection deficits in somatoform pain patients may therefore not be mere epiphenomena of depression. Of note, LEAS scores were not found to be associated with any other measure. This result replicates previous findings showing that LEAS sores are largely independent of BDI and TAS scores [10,24].

No significant associations between chronic pain-severity and any other measure were found, including quality of life. Finally, both BDI-depression and TAS-alexithymia showed a significant negative association with quality of life, which adds evidence to the notion that psychopathological rather than somatic processes may play the central role in somatoform pain.

Taken together, these results indicate that ToM abilities might be relevant determinants of quality of life in somatoform pain and a worthy target for future studies. Quasi-experimental studies in which mentalizing skills are trained are necessary to determine whether these findings can be utilized clinically.

### Sample

The present patient sample showed several characteristics typical for somatoform pain (see: Table 1). All patients suffered from pain although no adequate somatic explanation was detectable. Most patients reported chronic pain in more than one bodily domain, but also suffered from symptoms of depression. Most patients had a protracted medical history and consulted various practitioners, receiving various treatments. Accordingly, the use of analgesic and psychotropic medication in the patient group was omniprevalent. In contrast, the healthy control sample was free of chronic pain, as well as analgesic and psychotropic medication, by definition.

### Limitations

The cross-sectional design and particularities of our samples are the main limitation of our present study. No causal inferences should be drawn from cross-sectional studies: The observed impairments in emotional introspection may affect the etiology and maintenance of somatoform pain, or—to the contrary—be a consequence of ongoing pain. Further, systematic group differences may explain the observed results. For example, the observed mentalizing and emotional awareness deficits may be explained by the fact that literally all patients were receiving analgesic and/or psychotropic medication at the time of testing. Accordingly, we cannot rule out that the observed introspection deficits in somatoform pain patients are side-effects of analgesic and/or psychotropic medication [25].

Of note, we found that LEAS and FHAT scores correlated significantly with the length of participants’ responses. These correlations indicate that participants’ motivation and/or individual differences in verbal and literal expression capabilities are confounding factors for both testing instruments. Future studies with representative sample-sizes and adequate control-measures are needed to exclude that these confounds jeopardize the validity of the LEAS and the FHAT. However, the risk for confound by these factors is low for the present study, since both groups reached a similar word count on both scales.

## Conclusions

The present study provides evidence for deficits in mentalizing abilities and emotional awareness in chronic somatoform pain. Longitudinal studies are needed to determine whether these deficits play a causal or maintaining role in the etiology of the disease, or whether these deficits are epiphenomena related to ongoing pain and/or medication. Controlled trials are needed to examine whether therapeutic interventions tailored to improve patient’s introspection deficits provide additional therapeutic benefits when included in multimodal pain therapy.

## Supporting Information

S1 File Study data in .csv format. (CSV)
